# Holocentric chromosomes

**DOI:** 10.1371/journal.pgen.1008918

**Published:** 2020-07-30

**Authors:** Mauro Mandrioli, Gian Carlo Manicardi

**Affiliations:** Dipartimento di Scienze della Vita, Università di Modena e Reggio Emilia, Modena, Italy

## Abstract

Holocentric chromosomes possess multiple kinetochores along their length rather than the single centromere typical of other chromosomes [1]. They have been described for the first time in cytogenetic experiments dating from 1935 and, since this first observation, the term holocentric chromosome has referred to chromosomes that: i. lack the primary constriction corresponding to centromere observed in monocentric chromosomes [2]; ii. possess multiple kinetochores dispersed along the chromosomal axis so that microtubules bind to chromosomes along their entire length and move broadside to the pole from the metaphase plate [3]. These chromosomes are also termed holokinetic, because, during cell division, chromatids move apart in parallel and do not form the classical V-shaped figures typical of monocentric chromosomes [4–6]. Holocentric chromosomes evolved several times during both animal and plant evolution and are currently reported in about eight hundred diverse species, including plants, insects, arachnids and nematodes [7,8]. As a consequence of their diffuse kinetochores, holocentric chromosomes may stabilize chromosomal fragments favouring karyotype rearrangements [9,10]. However, holocentric chromosome may also present limitations to crossing over causing a restriction of the number of chiasma in bivalents [11] and may cause a restructuring of meiotic divisions resulting in an inverted meiosis [12].

## Evolution and structure of holocentric chromosomes

### Evolution of holocentric chromosomes

Holocentric chromosomes were described for the first time in 1935 to identify chromosomes with a diffuse kinetochore (or with a diffuse kinetochore activity) making these chromosomes able to bind to microtubules along their entire length. In the last decades, several studies assessed that the same behaviour during mitosis can be observed not only for holocentric/holokinetic chromosomes, but also for polykinetic chromosomes that contain numerous (but discrete) microtubule-binding sites, but the term “holocentric/holokinetic” is still used for both [1,5,7].

Before molecular methods became available, the presence of holocentric chromosomes was evaluated mostly using cytology and, considering that many species are difficult to study cytologically, it can be surmised that the true presence of holocentrism may be underestimated. In addition, there are several taxa, whose chromosomes are still uncharacterized, but their phylogenetic position suggests that they should have holocentric chromosomes [7,13]. The presence of holocentric chromosomes has been up till now assessed in about 800 species, including insects, plants, arachnids, and nematodes [1,5,7] suggesting that generally holocentric chromosomes originated by convergent evolution from ancestors possessing monocentric chromosomes. Interesting exceptions are represented by insects belonging to Oligoneoptera and Neoptera, whose monocentric chromosomes probably evolved from holocentric ancestor in two different and independent events [7]. Evidence of convergent evolution suggests that holocentrism is adaptive, but the specific conditions under which holocentrism provided a selective advantage seem to be diverse for different taxa [7,14]. Indeed, in phytophagous insects (such as aphids and lepidopterans) holocentrism could be related to the production by plants of compounds able to induce chromosomal breakages (clastogens), whereas in other cases, holocentrism allows facing DNA damage resulting from desiccation and/or other chromosome-breaking factors [14]. Despite these differences, holocentric chromosomes present intrinsic benefits since chromosomal mutations, such as fissions and fusions, are potentially neutral in holocentric chromosomes in respect to monocentric ones. However, the hypothesis of holocentrism as an anticlastogenic adaptation have to be more systematically tested, including both controlled laboratory experiments and field studies across clastogenic gradients and large-scale phylogenetic analyses [8]. At the same time, Nagaki et al. [15] proposed that holocentrism can be easily acquired during plant and animal evolution by a slight difference in the kinetochore origin. In particular, they hypothesized that if the direction of kinetochore origin turns by 90° and occurs along the chromosome axes up to the telomeric regions, it is possible to “generate” holocentric chromosomes without any further step.

### Structure of holocentric chromosomes

**Fig 1:**
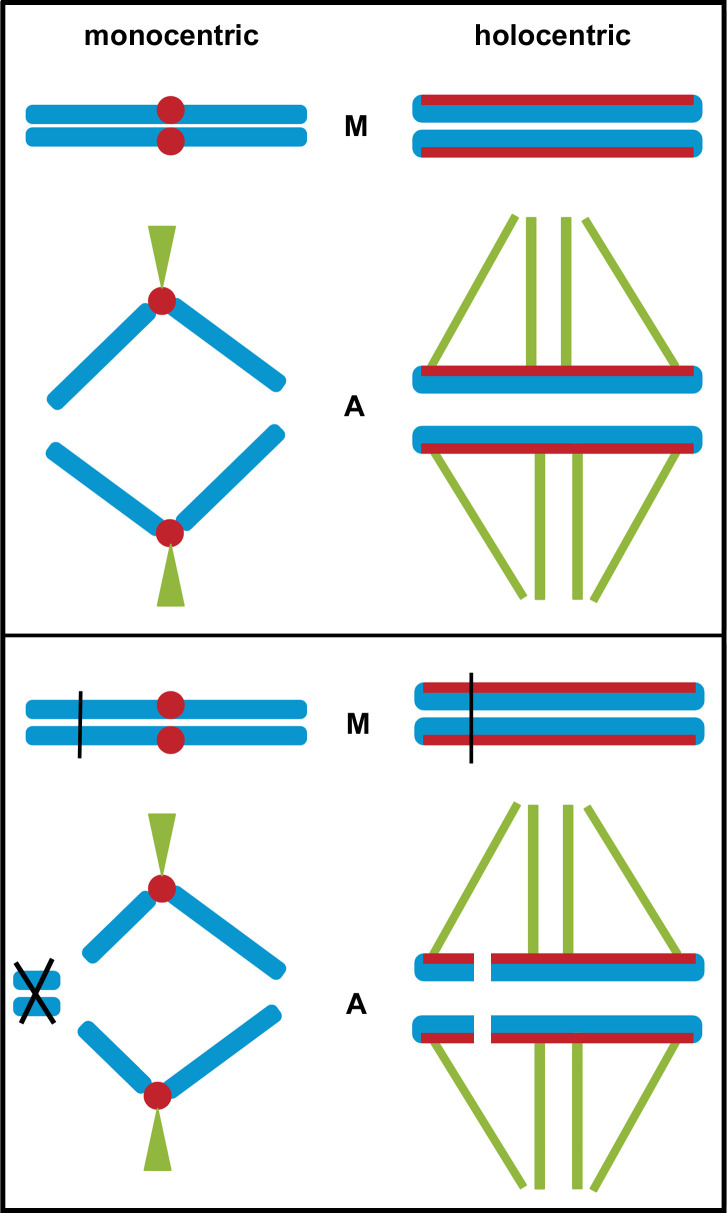
In monocentric chromosomes, kinetochore (red circles) can be easily identified as it is located at the chromosomal primary constriction (centromere) during metaphase (M, top box). At anaphase (A, top box) chromatids move towards poles after their attachment to microtubules and V-shaped structures can be observed resulting from the passive movement of the chromosomal arms. In holocentric chromosome a chromosome-wide kinetochore is present (red lines) and no primary constriction is present during metaphase (M, bottom box). During anaphase (A, top box) holocentric chromatids move towards poles as linear bars parallel. If a chromosomal breakage occurs in a monocentric chromosome (bottom box), acentric chromosome fragments cannot be attached to microtubules during metaphase (M) and they are lost during anaphase (A). On the contrary, chromosome breakage of a holocentric chromosome results in chromosomal fragments that retain kinetic activity due to the chromosome-wide centromere extension and can be properly inherited.

A detailed molecular analysis of the structure of holocentric chromosomes is currently available for the nematode *Caenorhabditis elegans* only [16,17], whereas the presence of true holokinetic nature has been also confirmed in other taxa by the evidence that experimentally induced chromosome fragments continue to attach to the spindle and segregate correctly [3]. For most of the species, data about holocentrism are related to the analysis of the behaviour of chromosomes during anaphase migration since holocentric sister chromatids migrate in parallel to the spindle poles, in contrast to monocentric ones in which pulling forces are exerted on a single chromosomal point and chromosome arms trail behind. As a consequence, chromatids of holocentric chromosomes move apart in parallel and do not form the classical V-shaped figures typical of monocentric ones [4] (Fig 1). Moreover, if a holocentric chromosome is fragmented (for instance by X-ray irradiation), each fragment retains centromere activity and can segregate properly to the poles.

## Holocentric chromosomes in arthropods

Among arthropods, the presence of holocentric chromosome has been reported in different species belonging to insects (Odonata, Zoraptera, Dermaptera, Psocoptera, Phthiraptera, Thysanoptera, Hemiptera, Trichoptera and Lepidoptera), scorpions (Buthoidea), mites and ticks of the superorder Acariformes and genus *Rhipicephalus* (Ixodidae), spiders (Dysderidae and Segestridae) [7,14], millipedes [18] and centipedes [18]. Despite this widespread occurrence, most of the currently available data on holocentrism is related to aphid and lepidopteran species [5,7]. In aphids, holocentric chromosomes have been deeply studied and their ability to stabilize chromosomal fragments has been associated to their phytophagous life style. Indeed, several plants produce chemicals able to induce DNA damage to pest insects. Nicotine, for instance, is a naturally occurring alkaloid found primarily in members of the solanaceous plant family (including *Nicotiana tabacum*) that can cause replication fork stress resulting in various forms of DNA damage, including chromosomal fragmentations [19,20]. Similar effects have been also reported by other plant-produced molecules, such as caffeine and ethanol [19,20]. In view of their ability to favour the inheritance of chromosomal fragments, holocentrism has been associated to recurrent changes in the karyotypes of some aphid species and in particular in the peach potato aphid *Myzus persicae*, where both inter- and intra-individual rearranged karyotypes have been also observed [21,22]. Interestingly, aphids also possess a constitutive expression of the telomerase coding gene so that they can initiate a *de novo* synthesis of telomere sequences at internal breakpoints, resulting in the stabilization of chromosomal fragments [23,24]. Among non-polyploid animals, Lepidoptera exhibit the highest variance in chromosome number between species within a genus and notable levels of interspecific and intraspecific karyotype variability [12,25,26]. Lepidoptera indeed tolerate chromosomal variations in view of their holokinetic chromosomes, which facilitate the successful inheritance of novel fission or fusion fragments. As a consequence, Lepidoptera can avoid the deleterious consequences of large-scale chromosomal fission and fusion [12,25,26]. Nevertheless, they can sometimes tolerate heterozygosity for multiple rearrangements in hybrids between population with differences in their karyotype, raising questions about additional mechanisms that rescue fertility in chromosomal hybrids. In Lepidoptera, therefore, chromosome evolution is believed to play a role in reinforcing speciation [12]. Comparing the genomes of lepidopteran species it has been also possible to analyse the effect of holocentrism in terms of rate of fixed chromosomal rearrangements. This approach evidenced in Lepidoptera two chromosome breaks per megabase of DNA per Million of years: a rate that is much higher than what observed in *Drosophila* and it is a direct consequence of the holocentric nature of the lepidopteran genomes [27,28]. At a structural level, insect holocentric chromosomes have not been studied in details, but it is interesting to underline the absence of homologues of CENP-C and CENP-A, previously considered essential for kinetochore functioning in eukaryotes [29].

## Holocentric chromosomes in nematodes

The best known group of holocentric species can be found in the Secernentea class of the nematodes, which includes *C*. *elegans* [16,17]. Other nematodes are usually described as holocentric because of their phylogenetic relationship to *C*. *elegans*, but real karyotypic evidences are scarce or controversial [30–32]. Nematode development is typically characterized by fixed lineages, therefore, it has been suggested that holocentrism could avoid the disastrous consequences of unrepaired chromosome breakage events [33]. The availability of several molecular and genomic resources allowed a detailed characterization of *C*. *elegans* holocentric chromosomes and in particular the structure of the kinetochore has been molecularly dissected [34,35]. Current data suggest that *C*. *elegans* kinetochores form paired lines or plates on opposite faces of condensed mitotic chromosomes [35], where each line represents the diffuse kinetochore of a single chromatid. Transmission electron microscopy of *C*. *elegans* chromosomes revealed that the kinetochore has a trilaminar structure very similar to that observed in monocentric chromosomes [35,36]. More than 30 different proteins have been identified as components of the *C*. *elegans* kinetochore and half of them was already known as functioning in the kinetochores of monocentric chromosomes. Among these, highly studied proteins include homologues of CENP-C and CENP-A, which are highly conserved structural component of the kinetochore in eukaryotes [36,37]. Contrarily to what generally observed in monocentric chromosomes, in holocentric ones the preferential localization of centromeres within heterochromatic areas is missing together with the presence of specific DNA sequences that in *C*. *elegans* are not required for the assembly of a functional kinetochore [36,37]. In this regard, it has been observed that holocentric chromosomes of nematodes are unique because they have a large number of satellites scattered throughout their genome, whereas no scattered satellites are found in the monocentric chromosomes of the nematode *Trichinella spiralis* [38]. Interestingly, these satellite DNAs are not conserved in their sequences among species suggesting that highly repetitive DNAs may facilitate the formation of kinetochores in view of their repetitiveness rather than for their specific sequence [38]. The absence of a localized centromere prompted several studies to identify proteins that are involved in the sister chromatid cohesion assessing that it is accomplished by a separate complex of conserved proteins, termed cohesin, that is comprised of the core subunits Scc3, Smc1, Smc3 and Scc1. Interestingly, they play the same function in organisms with monocentric chromosomes with an exception related to the subunit Scc1, whose gene in addition to the Scc1 orthologue present three additional paralogous genes [36,37].

## Holocentric chromosomes in plants

In plants, holocentric chromosomes have been found in zygnematophycean algae [39], in the genera *Myristica* (Myristicaceae), *Chionographis* (Melanthiaceae), *Cuscuta* (Convolvulaceae) and *Droseraceae* [40–42], in the species *Trithuria submersa* (Hydatellaceae), *Prionium serratum* (Thurniaceae) [43,44] and, among higher-plants, in many genera belonging to families Cyperaceae and Juncaceae, including the snowy woodrush *Luzula nivea* (Juncaceae), the most well-studied holocentric plant [45,46]. In *Luzula* spp, the centromeric activity is localized simultaneously at several evenly spaced sites along each chromosome and chromosomes can be fragmented naturally or by irradiation into smaller (but viable) chromosomes [47,48]. The presence of rearranged karyotype does not affect fitness, as assessed by studies reporting that *Luzula* hybrids with parents possessing smaller and larger chromosomes showed the smaller chromosomes aligned and paired with the larger ones [47]. Similarly, in plants belonging to the genus *Carex*, differentiation of the karyotype has been demonstrated to correlate with genetic divergence within species [49], among populations within species [50] and within populations [51] suggesting that, as previously reported in Lepidoptera [12], holocentric chromosome rearrangements contribute to genetic differentiation at different evolutionary scales in *Carex* evolution and speciation. In plants it has also been suggested that the diffuse kinetochore of holocentric chromosomes may suppress the meiotic drive of centromeric repeats and its negative consequences [46]. In particular, the expansions (or contractions) of centromeric repeats may lead to a larger (or smaller) kinetochore, which attracts more (or fewer) microtubules during meiosis [17,46]. This hypothesis, which correlates the presence of holocentric chromosomes with centromere drive suppression, is very intriguing but it only explains the evolution of chromosomal holocentrism in meiosis and not in mitosis and this is not trivial considering that some species with holocentric chromosomes may present a restriction of kinetochore activity during meiosis [17,52]. Similarly to what previously reported for *C*. *elegans*, in *L*. *elegans* centromeres are not made by centromere-associated retrotransposons nor centromere-associated satellite DNAs, but cenH3 proteins seem to be associated with a centromere-specific chromatin folding rather than with specific centromeric DNA sequences [53]. Conservation of elements between mono- and holocentric chromosomes is not limited to centromeric proteins, but it is also extended to epigenetic marks. Indeed, the cell cycle-dependent phosphorylation of serine 10 or serine 28 of histone H3 (that is typically enriched in peri-centromeric regions of monocentric plant chromosomes) occurs uniformly along the *Luzula* chromosomes [53]. As previously described in aphids, *L*. *elegans* possesses a rapid and efficient *de novo* telomere formation based on a telomerase-mediated healing process that is active immediately after chromosomal damage by irradiation of chromosomes [54]. Newly formed telomere repeats were cytologically detectable 21 days after irradiation in about 50% of cases with a complete healing of telomere after 3 months favouring the fragment stabilization and karyotype fixation [54].

## Holocentric chromosomes at meiosis: Unusual inverted meiosis to favour crossing over

**Fig 2:**
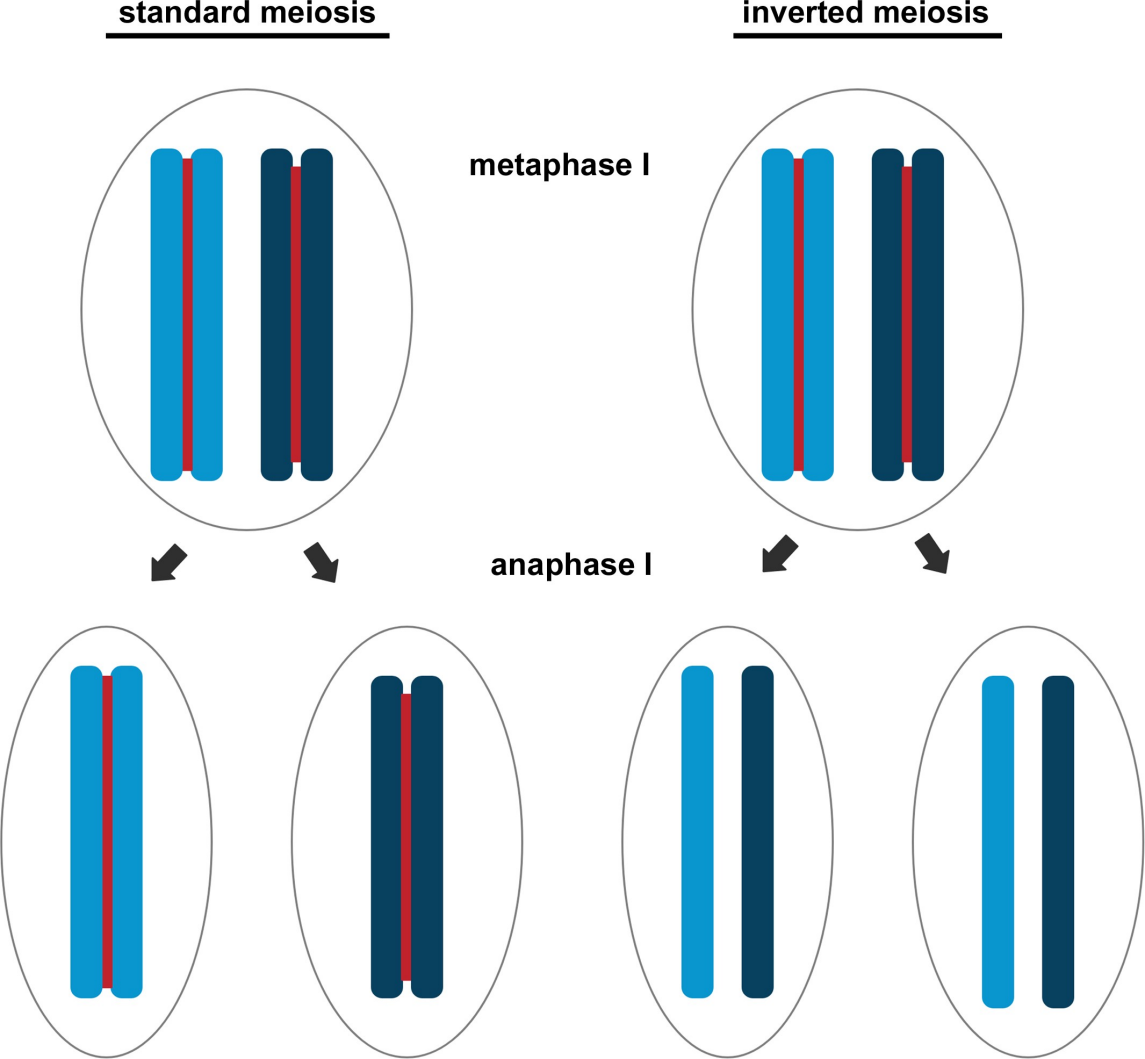
Schematic comparison of the chromosomal separation occurring during the first meiotic division in standard and inverted meiosis.

More than 120 years ago, van Beneden (1883) and Boveri (1890) described meiosis for the first time through a careful observation of germ cell formation in the nematode *Ascaris*. These observations, together with several further analyses, evidenced that canonical meiosis consists of a first division (called reductional division) that involves the segregation of chromosomal homologs resulting in the reduction of chromosome number and a second division (defined equational division) consisting in the separation of sister chromatids. A general rule for meiosis is therefore: first homologues, then sisters (see figure standard vs inverted meiosis) (Fig 2).

Ironically, the understanding of the reductional division in meiosis of *Ascaris* sp. has been obtained studying the holocentric chromosomes which, in many other taxa, follow a reverse order of meiotic division [12]. Indeed, as reported in several nematodes, in insects belonging to Hemiptera and Lepidoptera [55,56], in mites [57] and in some flowering plants [8] species with holocentric chromosomes generally present an inverted meiotic sequence, in which segregation of homologs is postponed until the second meiotic division. Furthermore, in most cases of inverted meiosis the absence of a canonical kinetochore structure has been observed, together with a restriction of the kinetic activity to the chromosomal ends [12,55,56]. These changes are related to the peculiar cohesion occurring in tetrads of the holocentric homologous chromosomes during meiosis that impose obstacles to the releases of chromosomes involved in multiple crossing over events [55–57]. In the holocentric chromosomes of *C*. *elegans* female meiosis [58], this problem is circumvented restricting crossing over to form only a single chiasma per bivalent and triggering the redistribution of kinetochore proteins along the bivalent axis forming meiosis-specific cup-like structures that uniformly coat each half bivalent but are excluded from the midbivalent region [58]. During anaphase I, *C*. *elegans* homologous chromosomes are segregated to the poles by microtubule pushing from the midbivalent regions towards the poles [58]. Differently to what reported in *C*. *elegans*, other organisms with holocentric chromosomes, including both plants and insects [12,55,56], circumvent this problem segregating sister chromatids during meiosis I leading to the term inverted meiosis in which the order of reductional and equational division is inverted in respect to canonical meiosis. In this case therefore the separation of homologous chromosomes follows the segregation of sister chromatids. However, in order to have a successful inverted meiosis, it is necessary that a bipolar orientation of sister kinetochores occurs, together with their attachment to microtubules from opposite spindle poles in meiosis I. This allows the segregation of sister chromatids to opposite poles in anaphase I (equational division), but it requests a mechanism to align and pair homologous chromosomes during the second meiotic division [55,56,57]. Interestingly, the presence of inverted meiosis can also facilitate the proper chromosome segregation in hybrids from parental species with differences in their karyotypes or derived by populations with rearranged karyotype allowing rescue of the fertility and viability of hybrids and promoting a fast karyotype evolution and possibly chromosomal speciation, as reported in Lepidoptera [12].

## Future directions

It is generally assumed that eukaryotic chromatin possesses some degree of compartimentalization so that the distribution of genes on monocentric chromosomes is generally non-uniform [59]. Conversely, the study of gene density in the spider mite *Tetranychus urticae* and in the nematode *C*. *elegans* revealed that genes are fairly constant distributed across chromosomes, although some differences are apparent in *C*. *elegans* between autosomes and the X chromosome, where genes are at a lower density and more evenly distributed [60,61]. Similarly, cytogenetic results suggested that in the aphid *Megoura viciae* the distribution of genes was uniform throughout all autosomes, with some differences related to X chromosomes where a certain degree of compartimentalization has been observed [62]. It could be therefore very intriguing to increase data related to gene mapping in species with holocentric chromosomes to confirm this diffuse gene distribution. Furthermore, the increasing availability of wholly sequenced genomes of organisms possessing holocentric chromosomes could help to shed light on the molecular machinery involved in the evolution of these peculiar chromosomes. Indeed, holocentric chromosomes evolved in multiple and independent events by convergent evolution [7]. Data from *C*. *elegans* clearly suggested that the functioning of centromere and kinetochore in nematodes is based on genes that were already known in monocentric organisms, such as HCP-1, HIM-10, ZW10, CENP-A and CENP-C [34]. The availability of genomics data could therefore allow to dissect at a genome-level the origin of holocentrism in order to better understand if the same genes (or different) have been coopted to favour the shift from mono- to holocentrism. Lastly, a more detailed study of the holocentric chromosomes evolution could be useful to understand which costs and advantages acted as main drivers in the evolution of the chromosome structure in order to better understand the multiple shifts from mono- to holocentrism (and vice versa) that occurred during both plant and animal evolution, even within recent lineages [63].

## Supporting information

S1 Text Version history of the text file. (XML)

S2 Text Peer reviews and response to reviews. (XML)
